# Structural outcomes in the Cleft Care UK study. Part 2: dento-facial outcomes

**DOI:** 10.1111/ocr.12109

**Published:** 2015-11-16

**Authors:** R Al-Ghatam, T E M Jones, A J Ireland, N E Atack, O Chawla, S Deacon, L Albery, A R M Cobb, J Cadogan, S Leary, A Waylen, A K Wills, B Richard, H Bella, A R Ness, J R Sandy

**Affiliations:** School of Oral and Dental Sciences, University of BristolBristol, UK; South West Cleft Team, University Hospitals Bristol NHS Trust, Cleft Lip and Palate TeamBristol, UK; Birmingham Children’s Hospital NHS TrustBirmingham, UK; National Institute for Health Research (NIHR) Biomedical Research Unit in Nutrition, Diet and Lifestyle at the University Hospitals Bristol NHS Foundation Trust and the University of BristolBristol, UK

**Keywords:** cleft lip, cleft palate, face, treatment outcome

## Abstract

**Objectives:**

To compare facial appearance and dento-alveolar relationship outcomes from the CSAG (1998) and CCUK (2013) studies.

**Setting and sample population:**

Five-year-olds born with non-syndromic unilateral cleft lip and palate. Those in the original CSAG were treated in a dispersed model of care with low-volume operators. Those in CCUK were treated in a more centralized, high-volume operator model.

**Materials and methods:**

We compared facial appearance using frontal view photographs (252 CCUK, 239 CSAG) and dental relationships using study models (198 CCUK, 223 CSAG). Facial appearance was scored by a panel of six assessors using a standardized and validated outcome tool. Dento-alveolar relationships were scored by two assessors using the 5-Year-Olds’ Index. Ordinal regression was used to compare results between surveys.

**Results:**

Excellent or good facial appearance was seen in 36.2% of CCUK compared with 31.9% in CSAG. In CCUK, 21.6% were rated as having poor or very poor facial appearance compared with 27.6% in CSAG. The percentage rated as having excellent or good dento-alveolar relationships was 53.0% in CCUK compared with 29.6% in CSAG. In CCUK, 19.2% were rated as having poor or very poor dento-alveolar relationships compared to 36.3% in CSAG. The odds ratios for improved outcome in CCUK compared to CSAG were 1.43 (95% CI 1.03, 1.97) for facial appearance and 2.29 (95% CI 1.47, 3.55) for dento-alveolar relationships.

**Conclusions:**

Facial and dento-alveolar outcomes were better in CCUK children compared to those in CSAG.

## Introduction

A child born with a cleft lip and/or palate (CLP) will receive treatments from a range of specialties as the anomaly affects several anatomical areas. Correction of anatomical structures is a key determinant of facial appearance, dento-alveolar relations and function. Appearance and dento-alveolar relationships are thus important outcome measures in these children.

A number of methods are available to evaluate appearance. These include photographs, videotapes, projected cine films, black and white drawings, identikit pictures (a composite picture made from individual elements) and computer generated pictures 1. Facial aesthetics can be determined by direct clinical assessment or indirect assessment using models or clinical photography 2. Much work has gone into developing measures for rating nasolabial aesthetics. Probably, the most widely adopted method is that described by Asher-McDade et al. 3. This method focuses on rating photographs of the child’s face and uses a five-point rating scale from very poor to very good. It can be used to assess nasal form, symmetry of the nose, shape of the vermillion border as well as upper lip and nasal profile. This outcome measure has been used in several large multicentre studies 4,5.

Several systems have been described for measuring dento-alveolar relationships as an outcome measure for CLP. The first widely adopted dento-alveolar outcome measure was the Great Ormond Street, London and Oslo Yardstick, more commonly known as GOSLON 6. The reliability of the yardstick has been confirmed in several studies 7–9. Although GOSLON provides a useful outcome measure in the early permanent dentition, it cannot be used to assess anatomical outcome until a child is at least 10 years old. A child of this age with cleft lip and palate may have had orthodontic treatment or secondary alveolar bone grafting that, along with primary surgery, will influence the observed outcome. The 5-Year-Olds’ Index was therefore developed to provide an earlier measure of outcome that is strongly determined by primary surgery 10.

Following CSAG, the 57 units which provided cleft care before have now been reduced to 11 centres or managed clinical networks 11. This has led to an increase in the number of children seen and treated in each cleft unit, and it is anticipated that this high-volume multidisciplinary service has driven up the quality of care and outcomes. The primary objective of this study was to compare two key anatomical outcomes for children born with UCLP (facial appearance and dento-alveolar relations) pre- and post-CSAG to assess potential changes in quality of care. The secondary objective was to assess agreement between and within assessors for both outcomes.

## Materials and methods

### Study design and sample

The data came from two cross-sectional studies in the UK, the CSAG 12 and CCUK 13. In both studies, we tried to recruit all children with non-syndromic UCLP over a defined period. The total sample size was 239 and 268 in the CSAG and CCUK study, respectively. Details of the recruitment and selection of children into these studies can be found elsewhere 12,13. The current analyses include 239 and 252 children with frontal facial photographs and 223 and 198 children with dental study models from the CSAG and CCUK groups, respectively.

### Photographs and rating

**Figure 1:**
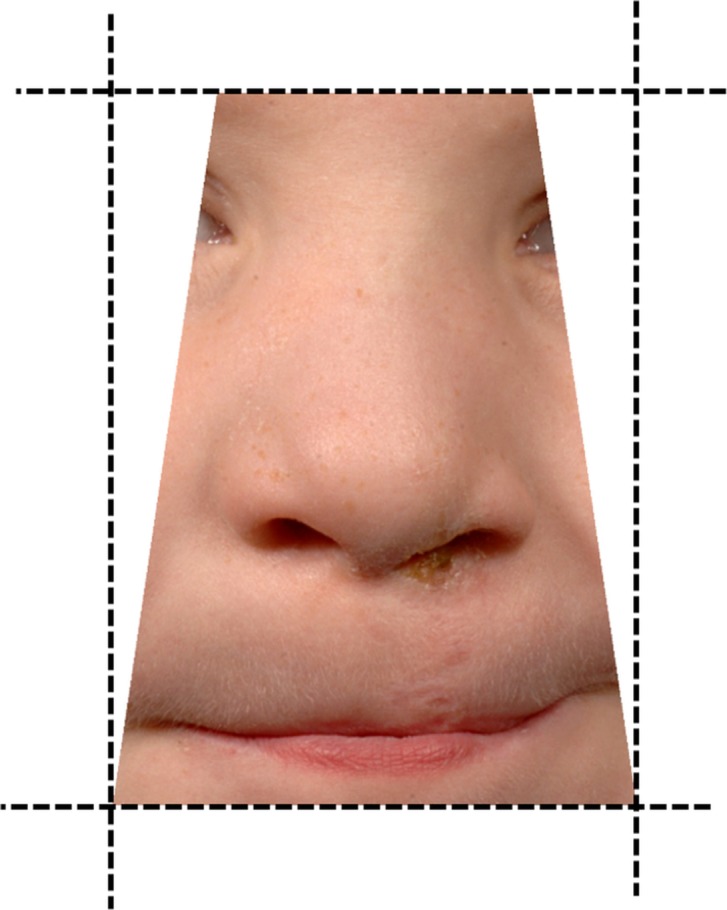
Photographs were cropped so that the final image in each case included both medial canthi, a small portion of sclera, all of the upper lip and lower lip and the oral commissures.

All of the photographic prints from CSAG were taken using a standardized protocol. 14. These were converted to digital images using a HP Photosmart 5515 e-ALL-IN-ONE scanner (Palo Alto, CA, USA). The photographs from CCUK were standardized using the recommended guidelines for audit photographs of children with cleft published in 2004 13,15,16. All of the CCUK photographs were taken by the cleft centre’s medical photography unit. To preserve the quality of the scanned (CSAG) and original digital (CCUK) images during cropping, Adobe Photoshop CS3 software (San Jose, CA, USA) was used to edit the images. All images were cropped using a trapezoid-shaped crop tool within Roxio PhotoSuite Version 9 software (Corel Corporation, Ottawa, Ontario, Canada) and the final image in each case included both medial canthi, a small portion of sclera, all of the upper lip and lower lip and the oral commissures. The iris of the eye and the hair were excluded (Fig.1).

There is no internationally accepted system for aesthetic assessment of individuals with cleft lip and palate 2. As a result, a team in the Birmingham Institute of Paediatric Plastic Surgery (BIPPS) have developed a standardized aesthetic outcome assessment tool for the evaluation of cleft lip and palate surgical repairs. This five-point ordinal scale was adapted from an existing method 17. In the screen shot of the cropped image, assessors were asked to rate the image as 1 = Excellent, 2 = Good, 3 = Fair, 4 = Poor or 5 = Very Poor. A collaboration agreement was negotiated with BIPPS that allowed us to use this web-based scoring tool.

The cropped and coded images were arranged for assessment, with a random number generator (http://stattrek.com/statistics/random-number-generator.aspx), ensuring there was no systematic clustering of images into CSAG or CCUK groups. The assessment of the collected images was made by a panel of six assessors comprising an adult with a cleft, a lay person and four cleft professional assessors from a single cleft unit (an orthodontist, a plastic surgeon, a clinical psychologist and a speech and language therapist). Each assessor was asked to rate a total of 491 frontal images (239 CSAG and 252 CCUK). After 4 weeks, 10% of randomly selected images from both the CSAG and CCUK were rescored. All of the data were then entered into an Excel spreadsheet.

### Study models and scoring

The collection of the CCUK study models is described in the methods paper in this supplement 13. All models were duplicated in white stone in a standardized format by a single technician who had also prepared the previous CSAG models. Models were categorized using the established 5-Year-Olds’ Index 10, again using a five-point ordinal scale. The models were categorized as either 1 = Excellent, 2 = Good, 3 = Fair, 4 = Poor or 5 = Very Poor. The assessment of the study models was made by two assessors: the first was a consultant orthodontist who originally designed the index and hence was experienced in its use; and the second was a consultant orthodontic trainee who had previous experience with the index. The study models were arranged in a random order (generated using www.ablebits.com) and were scored once by each assessor in 1 day. One week later, all the models were rearranged into a different random order and scored for a second time by both assessors. All of the data were then entered into an Excel spreadsheet.

The four scores for each of the CCUK study models (i.e. two assessors on two measurement occasions) were reduced to a single score for the main analysis. The coding rule was that if at least three of four assessment scores were the same, then that score was selected. If there was greater disagreement, then the study models were re-examined by both assessors together and a consensus reached. For CSAG, single scores (originally derived from four scores) were already available.

### Statistical analyses

For both photographs (CSAG and CCUK) and models (CCUK only), agreement between and within assessors was determined using Cohen’s weighted kappa (κ) statistic. Linear weights were used to allow comparability with previous studies 18, and in both cases for the five categories, the weights were 1.0, 0.75, 0.5, 0.25 and 0.0.

Comparisons between CSAG and CCUK were firstly made by combining photograph and model outcome categories into excellent (category 1), good, fair, poor (categories 2, 3 and 4) and very poor (category 5), then calculating the percentages of photographs and models in each category. For facial appearance, data from all assessors were combined to describe the distribution of categories in each survey. A mixed-effects ordinal logistic regression model was then used to compare the outcome in the CSAG vs. CCUK group using individual-level data from each observer. The mixed-effects model accounts for the fact that observations from each assessor were not independent. Ordinal logistic regression was also used to assess whether facial appearance had improved from the CSAG to CCUK group for each assessor individually. For dental relationships, an ordinal logistic regression model was used to compare the outcome in CSAG and CCUK using the single score from both groups. For all models, the outcome variables were reversed such that a higher score represented a better outcome. Hence, if the odds ratio was greater than one, then the odds of a better outcome were higher among children in the CCUK study vs. CSAG study. Data were analysed using Stata (version 12) http://www.stata.com/.

## Results

### Photographs

#### Agreement between and within assessors

Table1a,b shows the overall level of agreement when rating the frontal views in both the CSAG and CCUK photographs between (Table1a) and within (Table1b) the members of the assessment panel. Between assessors, the overall level of agreement of both the CSAG and CCUK photographs was fair (range of weighted kappa: 0.18–0.50 and 0.13–0.38, respectively). The strongest level of agreement for both the CSAG and CCUK photographs was between the orthodontist and speech and language therapist. The lowest agreement was between the lay person and the psychologist. Within assessors, the agreement of the pooled CSAG and CCUK photographs was fair to good (range of weighted kappa: 0.13–0.64). Within assessors, the speech and language therapist showed the strongest level of agreement and the lay person showed the lowest level of agreement with other assessors.

**Table 1:** Photographs: Agreement (a) between assessors in the CCUK and CSAG groups and (b) within assessors in the CSAG and CCUK groups – weighted kappa (95% CI)

Assessor	Patient	Ortho	SLT	Surgeon	Psych
(a)					
CCUK					
Lay	0.20 (0.13–0.27)	0.22 (0.14–0.31)	0.17 (0.10–0.24)	0.25 (0.17–0.33)	0.13 (0.04–0.21)
Patient		0.27 (0.18–0.35)	0.38 (0.29–0.47)	0.26 (0.18–0.34)	0.24 (0.16–0.32)
Ortho			0.38 (0.30–0.45)	0.34 (0.26–0.42)	0.23 (0.14–0.30)
SLT				0.24 (0.18–0.32)	0.22 (0.16–0.29)
Surgeon					0.24 (0.16–0.34)
Psych					
CSAG					
Lay	0.24 (0.15–0.32)	0.27 (0.18–0.36)	0.29 (0.21–0.38)	0.29 (0.20–0.38)	0.18 (0.10–0.25)
Patient		0.36 (0.29–0.44)	0.39 (0.30–0.47)	0.36 (0.28–0.45)	0.25 (0.18–0.32)
Ortho			0.50 (0.42–0.59)	0.37 (0.28–0.45)	0.32 (0.25–0.39)
SLT				0.36 (0.29–0.44)	0.23 (0.18–0.30)
Surgeon					0.29 (0.22–0.38)
Psych					

Assessor					Pooled frontal views CSAG and CCUK
(b)					
Lay					0.13 (0.07–0.24)
Patient					0.38 (0.21–0.55)
Ortho					0.56 (0.38–0.72)
SLT					0.64 (0.49–0.77)
Surgeon					0.48 (0.34–0.64)
Psych					0.23 (0.04–0.41)

Lay, layperson (architect); Patient, adult with a cleft; Ortho, orthodontist; SLT, speech and language therapist; Surgeon, cleft surgeon; Psych, psychologist.

#### Comparison between CSAG and CCUK

**Figure 2:**
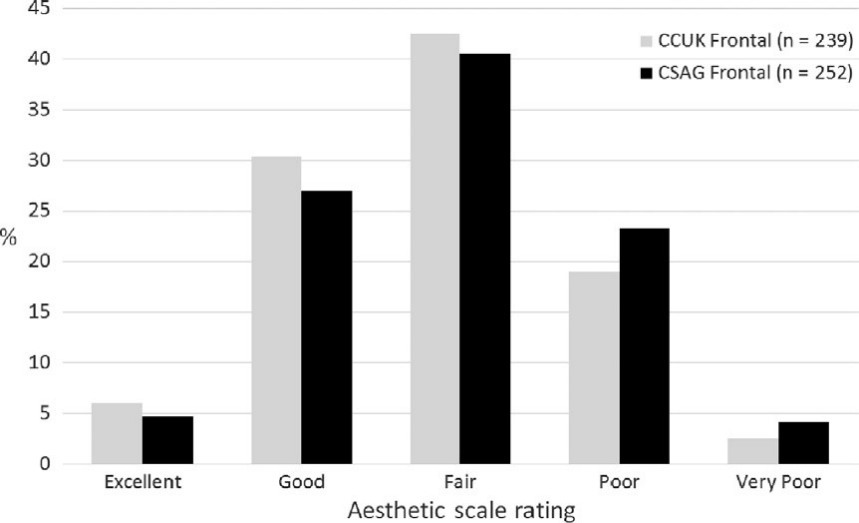
Categorization of photographic assessment of facial appearance from frontal views in the CCUK and CSAG groups, pooling data from the six observers.

Fig.2 shows the distribution of photographic scores in the CSAG and CCUK groups, pooling data from all observers. In the CCUK group, 36.2% had a good or excellent facial appearance compared to 31.9% in the CSAG group. The CCUK group had 21.6% poor or very poor facial appearances compared to 27.6% in the CSAG group. These findings indicate an improved outcome for facial appearance as measured from frontal views in the CCUK group compared to the CSAG group [odds ratio (of improved outcome in CCUK compared to CSAG) = 1.43; 95% CI: 1.03, 1.97; *p* = 0.032]. It should be noted that children in the CCUK study were on average 10 months younger than those in the CSAG study, and younger children tended to be rated with better facial appearance.

The odds ratios for a better outcome in CCUK compared to CSAG from each assessor are shown in Table2. Overall, the odds ratio shows that of the six assessors, four rated appearance as having improved in the CCUK sample compared with CSAG (the adult with a cleft, the orthodontist, the speech and language therapist and the psychologist). The layperson rated the CCUK outcomes as worse than CSAG, whereas the surgeon found there were no differences between CSAG and CCUK outcomes.

**Table 2:** Photographs: Odds ratios and 95% confidence intervals for having a better outcome in the CCUK group relative to the CSAG group (>1 indicates a better outcome for CCUK)

Assessor	OR (95% CI)
Lay	0.58 (0.41–0.81)
Patient	1.88 (1.36–2.61)
Ortho	1.64 (1.16–2.31)
SLT	1.41 (1.01–1.97)
Surgeon	1.04 (0.75–1.43)
Psych	1.98 (1.42–2.74)

Lay, layperson (architect); Patient, adult with a cleft; Ortho, orthodontist; SLT, speech and language therapist; Surgeon, cleft surgeon; Psych, psychologist.

### Study models

#### Agreement between and within assessors

Table3 shows substantial agreement between assessors and very good agreement within assessors for study model scoring.

**Table 3:** Study models: Agreements between and within assessors in CCUK – weighted kappa (95% confidence interval (CI) [Data were not available for agreement analysis on study models in CSAG]

	Weighted kappa (95% CI)
Interassessor agreement	
Scoring session 1	0.77 (0.71–0.82)
Scoring session 2	0.72 (0.66–0.78)
Intra-assessor agreement	
Consultant orthodontist	0.86 (0.81–0.90)
Consultant orthodontic trainee	0.83 (0.77–0.89)

#### Comparison between CSAG and CCUK

**Figure 3:**
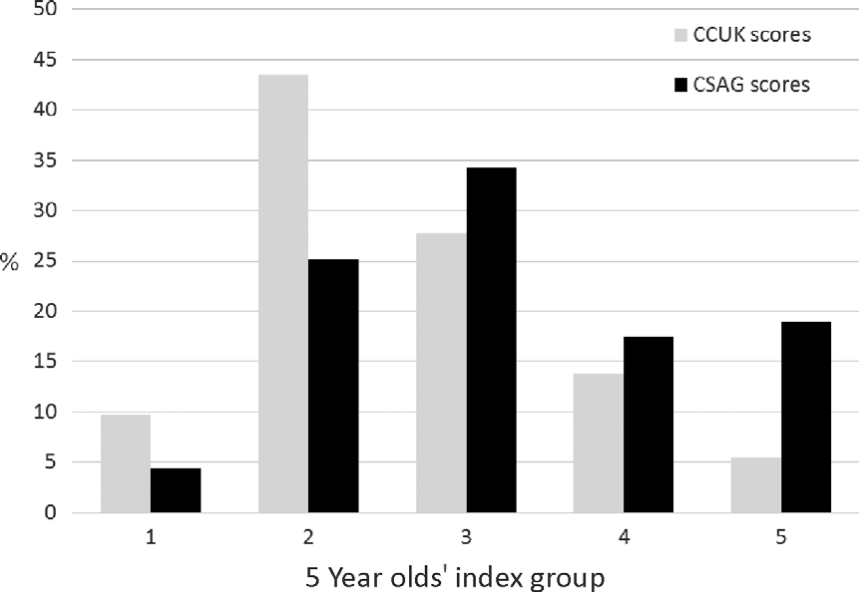
Categorization of assessment of dental relationships from the study models in CCUK and CSAG groups.

As seen in Fig.3, 53.0% in the CCUK group had good or excellent dental relationships compared to 29.6% in the CSAG group. In the CCUK group, 19.2% had poor or very poor dental relationships compared to 36.3% in the CSAG group. These findings suggest improved outcomes in CCUK compared to CSAG [odds ratio (of improved dental relationships in CCUK compared to CSAG) = 2.63 (1.85, 3.76), *p* < 0.001]. Data on age of children were available for 218/223 of the CSAG models and 197/198 of the CCUK models. When we adjusted for age, the odds ratio for a better outcome was moderately attenuated from 2.69 (95% CI: 1.85, 3.86) to 2.29 (95% CI: 1.47, 3.55) among those individuals with information on age. Unfortunately, we were not able to link the photographs to the age of the children and so were unable to examine whether age affected the difference we report between surveys for this finding.

## Discussion

This study repeated, as closely as possible, the original CSAG study which was conducted some 15 years ago. We have reported two outcomes (facial appearance and dento-alveolar relations) in 5-year-old children born with unilateral cleft lip and palate. In repeating the study, we needed to be confident that recruitment to both studies was similar and that the collection of photographs and models was not biased. The recruitment issues have been discussed in the first paper of this series 13. There were differences between CSAG and CCUK in the methodology and analysis to assess facial appearance from the photographs. Similarly, the model collection varied between the two studies. In the latter, key differences were that in the CCUK collection, there was an assumption that photographic images of the teeth could be used instead of models 19. The photographs were not always able to provide similar information to the models. Inter- and intrarater agreement was poor and we abandoned their use. There were therefore fewer models available in CCUK than CSAG and potentially those children whose models could not be obtained might have had worse outcomes. If this was the case, results would be biased in favour of better outcomes in CCUK. However, even if all those with photographs had outcomes that were fair, poor or very poor, the proportion of good or excellent outcomes in CCUK would be 42% which is still substantially better than CSAG (29.6%).

Although all efforts were made to ensure equivalent quality of facial photographs in both groups, such as the use of same resolution and dimension adjustments, some aspects could not be standardized. For example, the CSAG sample comprised photographic prints that were scanned to derive a digital format. Some loss of detail would have occurred during the scanning process. In addition, although a standardized photography protocol was applied to both groups, the equipment used differed. In the CSAG groups, photographs were taken using Pentax 35 mm film-based SLR Camera, which is less sophisticated than the cameras used in the CCUK group (Digital Nikon D3 and D700; Nikon Corp, Minato-ku, Tokyo). Moreover, there was a difference in the magnification used in the CSAG group (1:6) and the CCUK group (1:8). It has been shown that evaluation errors using digital photographs may result because of the magnification factor 20. All of the photographs in the CCUK group were taken using recommended guidelines for photography of cleft audit patients published in 2004 15,16. The poorer quality of the scanned CSAG group images may have had an impact on these assessments of facial appearance.

### Frontal photographic views in the CSAG group and the CCUK group

The agreement between the members of the assessment panel rating frontal views was fair in both the CSAG group and the CCUK group. There was heterogeneity in the assessment panel which included a lay person, an adult with a cleft and professionals (cleft orthodontist, cleft speech and language therapist, cleft surgeon, cleft psychologist). Elsewhere, similar groups have evaluated facial appearance in adults with repaired UCLP with no correlation between professional and lay assessments of nasolabial appearance, perhaps not surprisingly given that the evaluation of facial appearance on cropped photographs is not a task familiar to the lay person 21. Professionals consistently rate the appearance as being ‘better’ than lay assessors 22–24. They appear to focus on different features of the face compared to lay people. The latter are less satisfied with lip and nose aesthetics, and the relative positions of the lips seem to dominate their appreciation of facial aesthetics 23,25,26. The assessment of the frontal views in both the CSAG and CCUK groups showed that the strongest level of agreement was between the orthodontist and the speech and language therapist. The poorest agreement was between the lay person and the psychologist.

It is worth highlighting that in other intercentre studies 5,27, assessment of facial appearance from photographs is less sensitive than using dento-alveolar relations to discriminate between categorized outcomes.

The 1998 cleft service reorganization appears to have resulted in improved facial appearance of UK 5-year-olds born with complete unilateral cleft lip and palate. The measurement error indicated by the, at best, moderate intra- and interassessor agreement for photographs would likely bias our comparisons towards the null. The odds ratio for a better facial appearance in CCUK children is thus likely to be underestimated in these analyses. That said, younger children rate better for facial appearance and children in the CCUK study were on average 10 months younger than those in the CSAG study. In addition, in the CSAG study, analogue pictures were used and these were then scanned, which may have resulted in loss of detail in making these assessments. Furthermore, the intra- and interobserver agreement was weaker than for the observations on dento-alveolar relations.

### CSAG and CCUK dento-alveolar relationships agreement scores

In the original CSAG study, dento-alveolar relationships were determined with the 5-Year-Olds’ Index and the interassessor agreement (weighted kappa) was 0.94 14. Intra-assessor agreement was not reported. The available CSAG data are consensus scores, making kappa impossible to calculate between scoring sessions and assessors. However, interassessor agreement was likely to have been good considering the high between assessor agreements.

The improvements in outcomes for CCUK are clear when compared to CSAG. Over 50% of children included in the CCUK study model sample were recorded as having a good dento-alveolar relationship and <20% had a poor relationship. By contrast, <30% of the CSAG sample was assessed as good and over 36% were poor. As the outcomes were measured in children aged around 5 years old, there are relatively few treatment factors which could have an effect on the observed result. Primary surgery is usually carried out before the child is 1 year old and is often the only major intervention before 5 years of age. Both alveolar bone grafting and orthodontics are undertaken at a later age and would not account for the differences seen. Although facial growth patterns can influence results, there is no reason to believe that these would be different between the CSAG and CCUK groups. Furthermore, at 5 years of age facial, growth patterns are not fully expressed and would not distort the result of the primary surgery. This leaves primary surgery, used to correct the anatomical relationships, as the most likely cause of the differences in dento-alveolar relationships in the CSAG and CCUK samples. There have been no major changes in the surgical techniques used in the UK. If anything, the surgical techniques used currently are more similar across all centres and there is a smaller range of surgical techniques employed in UK centres than in the past 28.

Three main changes as a result of CSAG may account for the improvements in these anatomical outcomes. First, centralization of cleft care has increased the volume of cases that each surgeon treats per year. Seventeen of the 19 primary cleft surgeons in the UK met a target of operating on between 40 and 50 cases annually in 2009–2010 29, whereas only a single surgeon achieved this in the original CSAG 14. Second, cleft-specific training following the development of cleft surgical fellowships means that all newly appointed cleft surgeons have undergone training in a specialized cleft unit. Finally, there is now an established audit culture in the UK which 1) encourages sharing of results linking practice with outcome and 2) promotes critical and reflective practice among the teams. This leads to a greater awareness among clinicians of their own outcomes and those achieved by others leading to improvements in practice. Potentially, centralization and the establishment of an audit culture have driven more consistent and effective practice with standard operating procedures being used at the right age.

### Comparison between CCUK outcomes and other cleft outcome studies

The 5-year-old index has face validity and can predict outcomes in older children 30. In the CSAG report 31, the recommendations for service change aimed to reconfigure UK cleft services so that they were able to match to the better quality child outcomes that were reported by the European centres identified in the Eurocleft six-centre study (UCLP cases aged 8–10 years, 149 study casts). The two best centres reported that around 10% of cases fell into the two worst categories (groups 4 and 5) with a mean GOSLON score of 2.47 and 2.59 7. In the recent Americleft study, the best centre had 18% with poor outcomes in groups 4 and 5, with a reported mean index score of 2.63, and the worst centre had 61% of poor outcomes in groups 4 and 5, with a reported mean index score of 3.66 32.

With regard to improved outcomes of dento-alveolar relationships in children born with UCLP in the UK post-centralization, the national registry, the Craniofacial Anomalies Network, known as CRANE reported on 5-year-old study casts (364) between 2004 and 2007 33. The distribution in categories is very similar to CCUK, but the sampling was only approximately 60% of the available models and not all cleft centres contributed. Nearly 90% of the scores were externally validated. Direct comparisons are difficult and the use of a mean score for categorical data (rather than medians with confidence intervals) is not appropriate. If data are reported in this way, one assumes that those study models categorized in group 2 are twice as poor as group 1 which is not the case. Nevertheless, centralization of cleft services in the UK has in part achieved the aim of producing outcomes on a par with the best European centres.

## Conclusions

There is evidence from this study that outcomes of anatomical correction of children born with UCLP have improved since 1998 when they were still being treated in a dispersed model of care. There is strong evidence of improved outcomes from study models of children treated pre- and post-CSAG (CCUK) at the age of 5 years, and although overall facial appearance improved from CSAG to CCUK, the evidence is less convincing but is supportive of this improvement. Service reconfiguration seems to have improved anatomy and appearance, and this might form the basis to predict that this would translate into benefits in function and psychological adaptation. These issues are covered in subsequent papers in this supplement. The evidence reported here indicates that the centralization of cleft services in the UK has improved appearance outcomes for children born with a cleft.

## Clinical relevance

Centralization of cleft services in the UK has been ongoing for the last 15 years with the reduction of the number of cleft centres from 57 to 11. This process was predicated on a belief, and some evidence, that outcomes would be optimized in a model of high-volume operators and concentrated care. Two key outcomes in children born with a cleft are facial appearance and dento-alveolar relations. In the previous Clinical Standards Advisory Group study, these outcomes were poor. The implementation of centralized multidisciplinary care appears to have resulted in improved outcomes for dento-alveolar relations and facial appearance.
